# Radiologists can detect the ‘gist’ of breast cancer before any overt signs of cancer appear

**DOI:** 10.1038/s41598-018-26100-5

**Published:** 2018-06-07

**Authors:** Patrick C. Brennan, Ziba Gandomkar, Ernest U. Ekpo, Kriscia Tapia, Phuong D. Trieu, Sarah J. Lewis, Jeremy M. Wolfe, Karla K. Evans

**Affiliations:** 1 Image Optimisation and Perception, Faculty of Health Sciences, University of Sydney, Sydney, NSW Australia; 2 Visual Attention Lab, Harvard Medical School, Cambridge, MA USA; 3 Department of Psychology, University of York, Heslington York, UK

## Abstract

Radiologists can detect abnormality in mammograms at above-chance levels after a momentary glimpse of an image. The study investigated this instantaneous perception of an abnormality, known as a “gist” response, when 23 radiologists viewed prior mammograms of women that were reported as normal, but later diagnosed with breast cancer at subsequent screening. Five categories of cases were included: current cancer-containing mammograms, current mammograms of the normal breast contralateral to the cancer, prior mammograms of normal cases, prior mammograms with visible cancer signs in a breast from women who were initially reported as normal, but later diagnosed with breast cancer at subsequent screening in the same breast, and prior mammograms without any visible cancer signs from women labelled as initially normal but subsequently diagnosed with cancer. Our findings suggest that readers can distinguish patients who were diagnosed with cancer, from individuals without breast cancer (normal category), at above-chance levels based on a half-second glimpse of the mammogram even before any lesion becomes visible on the mammogram. Although 20 of the 23 radiologists demonstrated this ability, radiologists’ abilities for perceiving the gist of the abnormal varied between the readers and appeared to be linked to expertise. These results could have implications for identifying women of higher than average risk of a future malignancy event, thus impacting upon tailored screening strategies.

## Introduction

Breast cancer is the most common cancer, and a major cause of cancer-related deaths, among women worldwide^1^. Mammography screening has been shown to reduce breast cancer mortality through early detection and treatment^2–4^. However, it has been demonstrated that 5% to 30% of cancers are missed on screening mammography (i.e. false negative cases), with error rates increasing up to 50% depending on the type of lesion present on the mammogram, the age of the woman and the level of mammographic density^5–8^. Retrospective analyses of previous mammograms that were reported as normal, but from women later shown to have breast cancer from a subsequent screening, suggest two alternative situations: false negative cases which contain visible clues that a cancer might be present, or no apparent sign of cancer. However the rapidity of subsequently diagnosed cancers with some of the latter cases^9,10^, raise the question as to whether prior mammograms of women that were reported as normal and without any apparent signs that cancer is present could in fact contain information that indicates a heightened risk of a future malignant event. A gist methodology^11–14^, which has been used to good effect previously in radiology, may be one way of establishing if such information is present.

The gist of the scene refers to the perceptual information that an observer receives from a momentary glimpse of an image^15^. It is a remarkable characteristic of the human visual system, which helps us to intelligently allocate attention and access stored knowledge about elements of a new environment even if a complex scene is presented for only 20 milliseconds^15–17^. In the context of medical image interpretation, the gist response refers to instantaneous perception of features relating to an abnormality in an image. These might be ignored or overruled following more detailed image evaluation because the gist signal appears to be a global signal, spread across the image, and not merely a lucky glimpse of a localized lesion. It has been previously hypothesized that radiological visual search starts with a global or “holistic” response that establishes context, identifies gross deviations from known normal pattern, and initiates a sequence of foveal checking fixations^11^.

The Evans *et al*.^18^ gist signal, while perhaps related to other global signals, differs in that it does not have a local source in the image. Gist signals can provide both “what” and “where” information. A brief glance at a bookshelf will give you the gist of a collection of books. Perhaps the gist will include information about the type of books (paperbacks, textbooks, etc). This “what” gist will not provide information about where the eyes should be deployed though it can inform the viewer about the likelihood that a specific book is present (no romance novels on the textbook shelf). In contrast, a quick glimpse of a kitchen can be used to direct the eyes to likely locations of the toaster because the gist will contain spatial layout information that constrains the location of toasters. Works by Kundel and Nodine^11^ and Gale *et al*.^14^ on the information in brief presentation of medical images has focused on the second class of gist information. The gist information can constrain subsequent fixations. Evans *et al*. argued that they were looking at a gist signal that was different. It involved the extraction of image statistics across the entire image, permitting cases to be categorized as either normal or abnormal at above chance levels. However, their gist signal was not presumed to be useful in choosing the next deployment of the eyes. To test this hypothesis, in their experiment^18^, mammographic images containing subtle abnormalities were presented at a range of stimulus durations (250–2000 milliseconds). Radiologists were asked to rate the abnormality on a scale from 0 (confident normal) to 100 (confident abnormal) and then localize the abnormality on a subsequent image of the breast outline. Performance on the normal/abnormal classification was above-chance, but experts’ ability to localize these abnormalities on an outline of the breast was at chance levels. As radiologists’ ability to localize the abnormalities was at chance levels, it was concluded that image categorization was based on global properties of mammograms and not based on accidental attention to a localized target.

A subsequent study also showed that, if women had detectable cancer in one breast, experts could classify mammograms from the contralateral breast, with no visible signs of cancer, as normal or abnormal at an above-chance level even though no overt signs of cancer were visible in that breast^19^. This latter result highlights one aspect of the potential clinical relevance of the gist signal since it is well known that women who have cancer in one breast are more likely to have future cancer in the other breast^20^. It has also been shown that women recalled at screening by radiologists who *felt there was something wrong* but could not localise any cancer assessment, have a 1.4–6.6 fold increased risk of developing aggressive and larger cancers (≥20 mm) within 3–10 years compared to those who were not recalled^21,22^. This evidence suggests that a gist signal could be predictive of future cancer if it was visible in a mammogram with no visible overt signs of cancer.

One previous conference presentation provides evidence that a detectable gist signal is present in the prior exams of women who will eventually develop cancer^23^. In the experiment, bilateral mammograms were presented to the reader. As breast tumours are usually unilateral, the gist signal could be related to the bilateral asymmetry detected by the readers. Also only a small difference between the readers’ performance and the chance-level was observed. In the present paper, unilateral images were presented to the readers to investigate if any global image statistics, rather than bilateral asymmetry, contributed to the gist signal. Also, we investigated whether the gist signal varied across different lesion types, breast density categories, lesion locations, and lesion sizes. As the Evans *et al*.^18^ gist signal does not have any localized source in the image, we hypothesized the gist signal will not differ as a function of distance of the lesion from the center point of display, where the readers were instructed to look prior to flashing of the mammogram. If gist is not related to a lucky glimpse of a localized lesion, the recorded abnormality probabilities for the cases should be independent of distance.

In our data, expert radiologists have an above-chance performance in distinguishing prior mammographic images belonging to women who are diagnosed with breast cancer, even when those prior images contain no apparent visible signs that cancer exists. This means that some women who will be diagnosed with breast cancer in the future may be identified even before lesions become visible, large and more challenging to manage. By identifying women at high risk, a reliable gist signal could be used to tailor screening pathways to facilitate early detection of the disease and personalized medical strategies can be implemented^24,25^.

## Result

### Distribution of the gist response among different categories of mammograms

In our experiment, 23 Royal Australian & New Zealand College of Radiology (RANZCR) certified breast radiologists and breast physicians viewed 200 unilateral craniocaudial (CC) mammograms and gave a score to show whether they would recall the patient on a scale from 0 (confident normal) to 100 (confident abnormal). Each mammogram was presented for a half-second in the centre of the screen. This was then followed with a white mask, representing the breast area. To encourage the reader to look at the centre of screen, prior to the presentation of each mammogram, a cross symbol appeared in the centre of the screen for a half-second.

**Figure 1:**
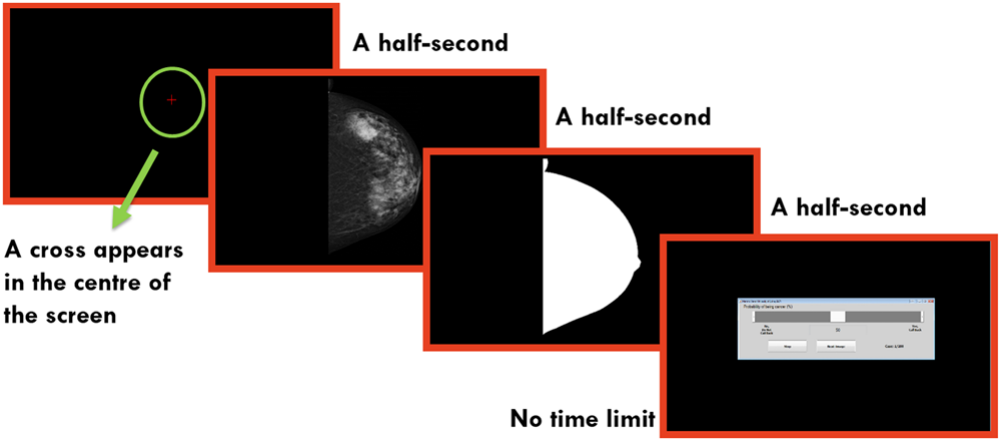
Experimental procedure.

The dataset included five categories of mammograms, with each category containing 40 cases. (Categories are further defined in the Methodology). The Cancer category contained current mammograms of women with biopsy-proven malignancy. The Prior-Vis cases were mammograms with retrospectively visible signs of cancer (Prior-Vis) but initially screened as normal. Contra cases were mammograms from normal breast contralateral to the malignant lesion. Prior-Invis cases were mammograms without visible signs of cancer from women who subsequently developed biopsy-proven malignancy. Finally, Normal cases were prior mammograms from women with no history of cancer at the time of the experiment and no cancer two years later. Figure 1 shows the experimental procedure.

**Figure 2:**
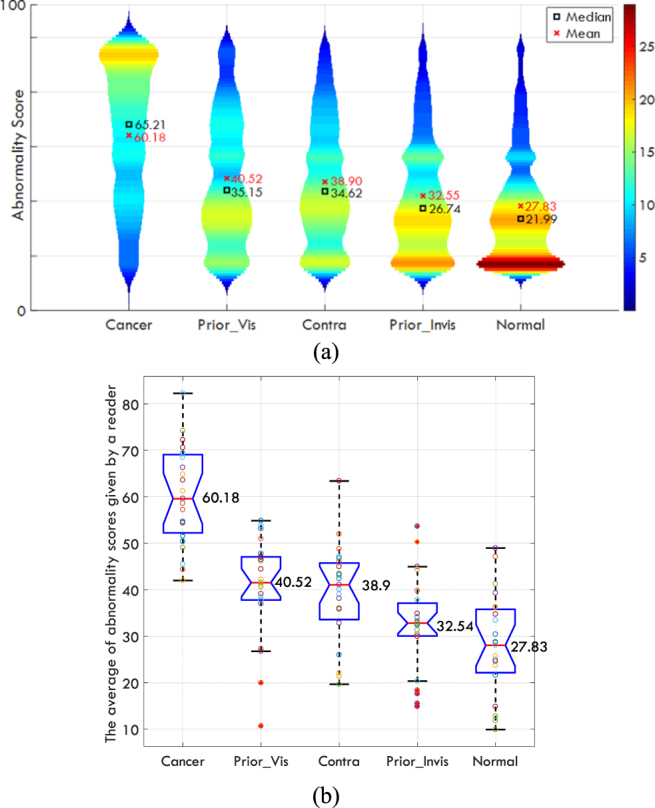
(**a**)Violin plot for all readings pooled together. In total, 4600 scores (23 readers × 5 categories × 40 cases per categories) were available. The color map shows number of images, the shape of plots indicates the probability density of the data across different categories. (**b**) The boxplot of the average of abnormality scores given by each reader to different categories (100 = maximum abnormality). Each circle shows a mean value of the given scores to mammograms in one category for one specific reader. The red line shows the median value and the notches shows the 95% confidence interval for the median value across the 23 individual radiologists’ values. The bottom and top edges of the box indicate the first and third quartile while the + symbol shows the outliers defined as any data point beyond 1.5 × the interquartile range (IQR). The whiskers are positioned at the start of this outlier position. The mean values for each category is written next to each boxplot.

There was no time limit for rating the mammograms, and the radiologists were not allowed to progress to the next image without first giving a rating. All five categories of mammograms were intermixed and presented using a randomly generated sequence. All participants completed the study in a single session of 25–40 minutes. We evaluated differences in abnormality across image categories using the Kruskal-Wallis H-test. The results suggested that each reader showed a significant difference among the categories (all p-values < 0.05) and the distribution of the average scores given by readers to different categories is shown in Fig. 2. Figure 2(a) shows the scores from all readings pooled together while Fig. 2(b) shows the distribution of average of abnormality scores given by each reader. Each circle shows the mean value of the given scores to mammograms in one category for each specific reader.

### Comparing radiologists’ ratings for each mammogram category against the normal category

**Figure 3:**
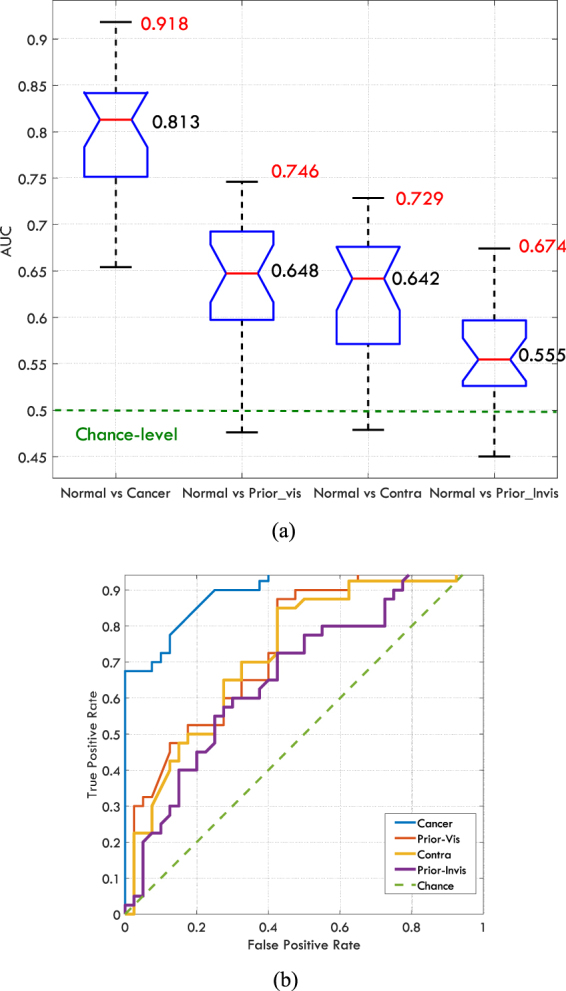
(**a**) The distribution of AUC values for each pairwise classification, the mean values, and the maximum AUC values are written in black and red respectively. (**b**) The ROC curves for the best reader across each pairwise comparison.

We evaluated whether the radiologists could distinguish mammograms in each of the positive categories (cases with abnormalities) from the normal mammograms using four pairwise classifications. To do so, for each pair, mammograms belonging to Normal category served as a baseline (Negative instances), and were combined with images from each of the four other categories (Positive instances). Therefore, for each radiologist, four pairs of analyses, which were Cancer vs Normal, Contra vs Normal, Prior-Vis vs Normal, and Prior-Invis vs Normal, were performed and hence each time 80 instances (2 × 40 images per category) were involved. Each time half of the mammograms were negative (Normal), whilst 40 images were exclusively from one of the 4 other categories described above. Receiver Operating Characteristic (ROC) curves, which shows the true positive rate (the proportion of positive instances correctly classified as such) against the false positive rate (the proportion of Normal images incorrectly classified as positive) at various cut-off points of the abnormality score, were created for each pair and for each radiologist. Area under ROC curves (AUC), were calculated for each paired grouping and each radiologist. AUC measures how well a reader can distinguish the Normal category from the other category in that pair. A value of 1 for AUC indicates a perfect classifier while a value of 0.5 represents a random classifier. Systematic over-rating or under-rating by a reader does not affect the AUC values. The AUC values are shown in Table 1, the distribution of AUC values from different readers is shown in Fig. 3(a). The ROC curves for the radiologist performed the best in each comparison are demonstrated in Fig. 3(b).

**Table 1:** The AUC values for the pairwise classifications for each observer, the Normal category served as the baseline.

	Normal vs			
	Cancer	Prior-Vis	Contra	Prior-Invis
1	0.711	0.476	0.568	0.517
2	0.729	0.695	0.622	0.529
3	0.850	0.653	0.670	0.544
4	0.824	0.719	0.675	0.652
5	0.829	0.726	0.700	0.585
6	0.739	0.597	0.642	0.598
7	0.803	0.659	0.608	0.525
8	0.813	0.638	0.549	0.575
9	0.876	0.695	0.677	0.555
10	0.840	0.720	0.680	0.561
11	0.778	0.587	0.617	0.484
12	0.779	0.641	0.567	0.522
13	0.769	0.563	0.670	0.574
14	0.869	0.654	0.687	**0**.**674**
15	0.825	0.648	0.657	0.610
16	**0**.**918**	0.686	0.690	0.602
17	0.815	**0**.**746**	**0**.**729**	0.605
18	0.843	0.648	0.670	0.550
19	0.746	0.573	0.561	0.544
20	0.654	0.495	0.479	0.450
21	0.733	0.628	0.550	0.537
22	0.865	0.644	0.633	0.593
23	0.791	0.598	0.584	0.471

The highest value for each pair is bolded.

To examine whether the AUC estimate for the readers was different from chance level (i.e. 0.5), Wilcoxon Signed Rank test was used. A p-value < 0.05 for the test indicates that the average reader can distinguish cases from that category from normal ones at above-chance level based on gist responses. We found that the AUC values were above-chance for each of the four categories: Cancer category (Signed rank = 276, z = 4.20, p < 0.001); Contra category (Signed rank = 275, z = 4.17, p < 0.001); Prior-Vis (Signed rank = 273, z = 4.11, p < 0.001); Prior-Invis (Signed rank = 260, z = 3.71, p < 0.001). The obtained results suggest that the average reader is able to distinguish patients who were diagnosed with the cancer from individuals without breast cancer at above-chance level based on half-second glimpse of mammogram even before the lesion becomes apparently visible (Prior-Invis).

**Figure 4:**
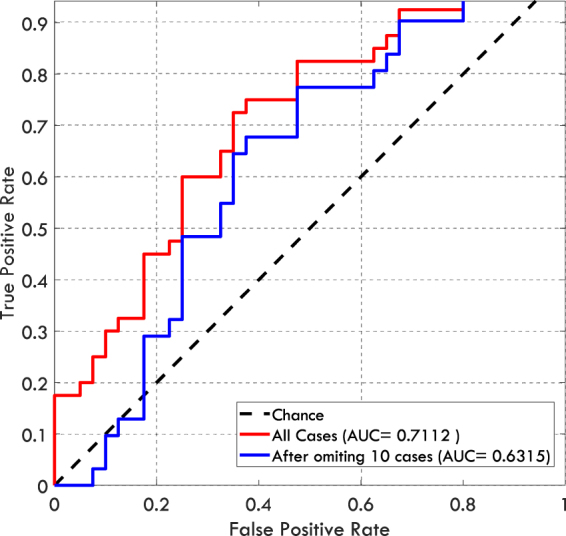
The ROC curves corresponding to the mean gist response calculated based on abnormality scores given by radiologists number 14 and 4 (the best and the second best readers). The red curve shows the classification performance when all cases in the Prior_Invis category were included and the blue curve shows the classification performance when ten cases in the Prior_Invis category with the highest abnormality scores were omitted. At this point the difference between the Prior_Invis and Normal categories is marginally significant.

The differences between the median of AUC values and the chance-level (0.5) were 0.313, 0.148, 0.142, and 0.055 for Cancer, Contra, Prior-Vis, and Prior-Invis categories respectively. Therefore, the gist signal is quite small for Prior-Invis category. Although the test result showed significant p-values for this category, one might argue that the difference between the median AUC for Prior-Invis category and the chance-level, was driven by one or two specific cases that deviated considerably from the normal appearance and by omitting them the difference would become insignificant. However, in three different ways, we show that the significant result we observe is not due to a small number of specific cases but is the characteristic of the whole image set in Prior_Invis category. Firstly, even after omitting two cases (5% of cases in the category), that gave the highest average scores, the difference between the median of AUC values and chance-level remained significant for the Prior-Invis category (Signed rank = 233, z = 2.89, p = 0.0039). Secondly, the mean gist response based on the average of abnormality scores given by the two best readers (radiologist number 14 and 4) to each case was considered. The mean response resulted in an AUC of 0.7112 and only after omitting 23 images in the Prior-Invis category the AUC dropped to a value less than chance-level (0.5). The omitted images were chosen by ranking all of the 40 images by their abnormality scores in the Prior-Invis category and omitting the first 23 with highest score. We included scores from two best readers rather than only the best one, to make sure that the observed results are not a random phenomenon in a single reading. Thirdly, the difference between the median of abnormality scores assigned to Normal and the Prior_Invis categories was compared using Mann-Whitney U-test. Before omitting any cases, the difference between the medians differed significantly (Rank sum = 1958, z = 3.25, p = 0.0012) and it remained significant up to the point that 10 images with the highest scores were omitted (Rank sum = 1279, z = 1.884, p = 0.0596). The ROC curves for the group average gist response are shown in Fig. 4.

One might also argue that the radiologists rated the mammograms based on the perceived density, however, the percentage density calculated^26^ did not differ significantly between the Normal category and the other four categories: Cancer category (p = 0.27); Contra category (p = 0.70); Prior-Vis (p = 0.79); Prior-Invis p = 0.62).

A Pearson correlation analysis demonstrated significant correlation between the radiologists’ AUC scores (Table 1) derived from the Prior-Invis vs Normal comparison with the scores derived from the Cancer vs Normal (p = 0.003; r = 0.59), Contra vs Normal (p = 0.007; r = 0.55), Prior-Vis vs Normal (p = 0.0004; r = 0.68) comparisons. Although the radiologists’ capabilities for perceiving the gist of the abnormal vary across the readers and also across different categories, this correlation analysis suggests that those radiologists who are better at detecting the gist signal in the prior images without overt signs were also better at distinguishing between abnormal and normal cases in each of the other comparisons. This hypothesis was endorsed by conducting a confirmatory factor analysis with one common factor (χ^2^(2) = 0.39, p = 0.82). The nonsignificant p-value indicates that there is no difference between the pattern of observed data presented in Table 1 (each reader served as an observation with four variables) and the fitted model. The interpretation of this unidimensional fit to data is that a radiologist could be viewed in terms of her/ his “overall capability for perceiving the gist of the abnormal” rather than her/his ability in perceiving gist in certain individual pairwise comparisons. The factor loadings and the specific variances are shown in Table 2.

**Table 2:** The factor loadings and the specific variances based on factor analysis with one common factor for the data presented in Table 1.

Variables	Factor loadings	Specific variances
AUC_Normal vs Cancer_	0.821	0.326
AUC_Normal vs Priro_Vis_	0.792	0.372
AUC_Normal vs Contra_	0.901	0.188
AUC_Normal vs Priro_Invis_	0.732	0.464

We also investigated if the probability given by the readers depends on lesion type, case density, lesion position, lesion size, and lesion distance from the center of display (i.e. where the cross presented before viewing each image). First, we examined whether images of different categories have comparable characteristics. Table 3 shows the characteristics of images included in different categories. For Prior_Vis, Contra, and Prior_Invis categories the lesion characteristics were assessed based on the screening mammograms, on which cancer was detected. The extended Fisher test was used to make sure that the proportions of cases with different BIRADS density categories, lesion types, and lesion locations were comparable among different categories of images. As shown all three p-values were insignificant, hence, the images of different categories were matched in terms of breast density, lesion types and locations. The Kruskal Wallis H-test was used to examine whether the lesion size and lesion distance from the center point are comparable among different categories. As shown in Table 3, both p-values were insignificant suggesting that the distribution of lesion size and distance from the center point were quite similar in different categories.

**Table 3:** The characteristics of cases in five categories.

	Cancer	Prior_Vis	Contra	Prior_Invis	Normal
Density					
BIRADS I (Fatty)	4	8	3	5	8
BIRADS II (Scattered fibroglandular)	17	20	25	19	17
BIRADS III (Heterogeneously dense)	19	11	12	11	14
BIRADS IV (Extremely dense)	0	1	0	5	1
p-value^*^	**0**.**112**				
Location					
Central	8	6	8	2	—
Inner	13	9	13	11	—
Outer	18	23	18	24	—
Retro areolar	1	2	1	3	—
p-value^*^	**0**.**482**				
Lesion type					
Architectural Distortion	2	2	2	1	—
Calcification	5	6	5	8	—
Discrete Mass	6	11	7	4	—
Non-specific density	11	5	11	7	—
Speculated Mass	5	6	5	8	—
Stellate	11	10	10	12	—
p-value^*^	**0**.**853**				
Lesion size (mm^2^)					
Mean	10.62	12.32	10.21	11.72	—
Min	3	3	3	5	—
Max	26	40	25	65	—
p-value^**^	**0**.**593**				
Lesion distance from the center point (pixels)					
Mean	1513	1421	1597	1406	—
Min	369	290	451	532	—
Max	3826	3904	3812	3751	—
p-value^**^	**0**.**912**				

^*^P-value from the extended Fisher’s Exact Probability test to evaluate if the distribution of cases differed among five categories of images.

^**^P-value from the Kruskal Wallis H-test to evaluate if the distribution of cases varied across five categories of images.

To evaluate if gist response varied as a function of lesion characteristics, again the mean gist response based on the average of abnormality scores given by the two best readers was considered. Similar to the previous analysis, we did so to make sure random noises from a single reading would not affect the results. Also, as discussed the radiologists’ capabilities for perceiving the gist of the abnormal vary. Hence, we restricted the analysis to high-performing readers. For each category of abnormal cases, i.e. Cancer, Prior_Vis, Contra, and Prior_Invis, by utilizing Kruskal Wallis H-test, we investigated if the mean gist response (probability of abnormality) varied across different BIRADS density categories, different lesion types, and different lesion position. Table 4 indicates the obtained results. As shown, only for Cancer category, the gist response differed significantly among different locations (p = 0.0462). However, after correction for multiple comparisons, the obtained p-value did not remain significant. In addition to the 4-scale BIRADS density categorization, we grouped images either as low (BIRADS I and II) or high (BIRADS III and IV) density and repeated analysis for the binary breast density score so that more samples per each category became available. Also, to investigate if the higher gist response is associated with the presence of mass or not, we split cases as mass and non-mass (calcification and architectural distortion) and ran the Kruskal Wallis test. We also repeated the tests when four abnormal categories were grouped together. None of the p-values were significant.

**Table 4:** Results of Kruskal Wallis H-test (χ^2^, p-value) for investigating whether the mean gist response differs as a function of breast density, lesion type, lesion position, lesion size, and lesion distance to the center point.

(χ^2^, p-value)	Density	Type	Position	Low/High density^*^	Mass/others^**^	Lesion size^¤^	Distance^§^
Cancer	4.46, 0.11	6.08, 0.30	**7**.**99**, **0**.**05**	1.45, 0.23	0.59, 0.44	0.54, 0.46	0.03, 0.86
Prior_Vis	4.82, 0.19	4.25, 0.51	0.59, 0.90	0.89, 0.34	0.04, 0.84	3.22, 0.07	0.49, 0.49
Contra	0.89, 0.64	5.82, 0.32	2.61, 0.46	0.89, 0.34	1.34, 0.25	0.00, 0.95	0.38, 0.54
Prior_Invis	0.63, 0.89	7.93, 0.16	1.08, 0.78	0.22, 0.64	0.06, 0.81	0.47, 0.49	1.83, 0.18
All abnormal	1.88, 0.60	1.66, 0.89	1.68, 0.64	0.18, 0.67	0.00, 0.97	0.10, 0.75	3.09, 0.08

^*^The low density group contained BIRADS I and II while the high density one comprised on BIRADS III and IV.

^**^Calcification and architectural distortion were grouped as “others”.

^**¤**^Lesions were divided into two groups, those larger than 10 mm^2^ (the median value) and those smaller than that.

^**§**^Lesions were divided into two groups, those further than 1343 pixels (the median value) and those nearer than that.

The significant p-value is shown in bold. However these p-values are not corrected for multiple comparisons.

Further we investigated if the lesion size and its distance to the center point were related to the strength of gist response. Unlike BIRADS density categories, lesion types, and lesion position, these two variables are continuous. Therefore, based on size of lesions, we categorized the images into two categories, one containing the larger lesions while the other included the smaller size lesions. The median lesion size was served as the threshold for the categorization. Similarly, images were also split into two groups based on whether the lesion distance to the center point was higher or lower than the median distance. Similar to the above-mentioned categorical variables, Kruskal Wallis H-test was employed. The obtained results are shown in Table 4. To ensure that the categorization of variables did not eliminate the dependency between two variables, we also used the Hilbert-Schmidt Independence Criterion (HSIC) for testing the independence of the mean gist responses and the lesion size as well as lesion distance to the center point. The HSIC measure the dependence between two random variables based on the fact that they are independent if and only if any bounded continuous function is uncorrelated. The HSIC test using a bootstrap approximation for the test threshold suggested that the mean gist response was independent from both lesion size and lesion distance for all four abnormal categories (i.e. Cancer, Priro_Vis, Contra, and Prior_Invis).

## Discussion

Screening mammography has been the frontline screening tool for breast cancer and has been credited with 30–40% reduction in deaths from the disease^2–4^. However, a range of between 5–50% of cancers are missed on screening^5–8^ and some women develop interval cancers. Our results indicated that based on a momentary glance (gist), radiologists can distinguish some mammograms of women who were reported as normal but diagnosed two years later with breast cancer at subsequent screening, from mammograms arising from women who have never been reported with breast cancer at above-chance levels. Here, we also replicate the Evans *et al*. 2016 findings of radiologist being able to detect cancer in a breast with no overt signs of cancer (diagnosed as normal) but that is the contralateral breast to the breast that has a visible abnormality. Interestingly, our results showed that even if no visible cancer signs are present in the mammogram, the radiologists’ performances were still above-chance. This is evidence by prior mammograms with no classic, overt signs of cancer can contain important information that may predict a future malignancy.

We also investigated if the gist response differed as a function of BIRADS breast density, lesion type, location, size, and its distance to the center point (Table 3). The results suggested that the gist response was independent from the lesion characteristics. This supports the Evans *et al*.^18^ hypothesis that gist signal, measured in experiments of this sort, does not have any localized source in the image. If the gist signal was related to a lucky glimpse of a localized target, the abnormality probabilities should have been higher for the larger lesions and/or those located toward the center of images. Obtaining a gist signal independent from these two variables further supports the Evans *et al*.^18^ hypothesis that the gist is based on some implicit extraction of statistics across the entire image and differs from the information that might be used to guide future eye movements for lesion detection.

At present, this gist information is not being utilized in any way. In principle, it would seem to have some clinical potential, however, making use of this gist information presents several challenges. Firstly, under current reporting procedures, an initially abnormal gist would be considered to be overruled if subsequent, more detailed evaluation did not reveal visible signs of cancer. We have known for many years that the initial appearance of a stimulus can serve to guide attention to the locus of a visible lesion. The gist signal, described here, is somewhat different. It is a global indication of an elevated risk of cancer, now or in the future. It is not pointing toward a specific location but appears to be a signal, diffused across the breast parenchyma. It might be used as an imaging biomarker or a warning sign but not a localizing signal. Thus, in order to be useful, the gist would need to be treated as an independent marker for future risk even if the current images show no cancer.

The second challenge is that the gist signal is quite small, especially in the interesting case where the visible cancer will not develop for several years. An important direction for future research would be to develop ways to increase the size of that signal. This could involve training an artificial intelligence tool to detect the signal and/or to train radiologists to detect the signal more reliably. Such work may tell us that some images are making a disproportionate contribution to gist. Knowing this would help us to isolate the critical components within images that give rise to gist impressions. This information will enable us to identify evidence-driven strategies to best identify the women for whom the gist is useful.

The third challenge concerns expertise. In this study, 18 of 23 radiologists read more than 2000 cases per year, which means they meet the minimum annual number of mammograms read suggested by the National Accreditation Standards in Australia^27^. All were registered as BreastScreen reading radiologists. Thus we do not know if the results would hold for observers who read fewer mammograms annually. Correlation analysis suggests that those readers who were best at differentiating between cancers and normal for the Normal vs Cancer comparison (and the other comparisons) were also best with the Normal vs Prior-Invis comparison, implying that the highest performing radiologists also have the strongest ability to detect the gist of abnormality. This implies that reader experience and expertise have important impacts upon gist detection but further research is required to understand what combination of general experience and specific expertise will produce good gist detection.

Our study has number of limitations. First, we only included CC view mammograms to avoid the impact of difference in mammographic view on the gist response. In the future, studying the gist responses on a larger set of images which comprised of both mediolateral oblique (MLO) and CC projections and investigating the impact of the view and radiologists’ capabilities to perceive the gist of the abnormal will be performed. Second, in the factor analysis we only considered radiologists’ performance in assessing different pairs of mammograms and concluded that generally some radiologists are better at detecting the gist signal no matter what the task was. However, as all four considered comparisons, i.e. Cancer vs Normal, Contra vs Normal, Prior-Vis vs Normal, and Prior-Invis vs Normal, dealt with lesion detection in mammograms, further experiments on other imaging modalities is required to assure that a unidimensional fitted model is sufficient to explain the radiologists’ capability for perceiving the gist of the abnormal. Thirdly, all prior images included in our dataset were acquired two years before the cancer detection, however, prior mammograms acquired greater than two years before might also contain information that indicates a heightened risk of a future malignancy and hence might result in a different gist response.

Finally, this study suggests that the gist of the abnormal has the potential to predict future malignant events. However, further investigations are required to find a proper way to utilize this phenomenon. One possible way would be to flash a case for a half-second and record the gist response, *before* usual presentation of case, and use this signal to predict future breast cancer. Also, the results showed that high gist response correlates with the cancer in the current image. Therefore, when a case is classified as abnormal based on the gist response but reported as normal after usual presentation of image, it might be cost-effective to send it to a second reader for double reading. Therefore, investigating the cost-effectiveness of different applications of the gist response and also finding an optimal way for recording the response could be a possible avenue for future work.

In summary, there is a detectable signal of abnormality present in mammograms with no overt signs of cancer. This signal is present in images taken years before the cancer develops. However, its use as an imaging biomarker will require further investigation to bolster the signal and develop its clinically warranted uses.

## Method

### Ethics approval

Institutional ethics approval was obtained from the Human Research Ethics Committees at the University of Sydney. Informed consent was obtained from all participants prior to any readings. All mammograms used in the study were de-identified. The NSW Cancer Institute, under a Memorandum of Understanding between Cancer Institute NSW and the University of Sydney, approved the access, use and publication of data originating from the BreastScreen NSW program and the BreastScreen Reader Assessment Strategy (BREAST) program. All methods were performed in accordance with the relevant guidelines and regulations of the Human Research Ethics Committees at the University of Sydney and NSW Cancer Institute.

### Mammographic images

The dataset contained 200 single breast craniocaudial mammograms that were acquired by digital technology and de-identified after being collected from the BreastScreen New South Wales Digital Breast Image Library. Malignant cases were biopsy proven while negative cases were assessed by the consensus reading of at least two senior radiologists following the negative screen reports of two clinical radiologists. The dataset included five categories of mammograms:

1. Cancer: Current mammograms of women (n = 40) with visible signs of cancer that were confirmed to be malignant through biopsy;
2. Prior-Vis: Prior mammograms of women (n = 40) with visible signs of cancer that were reported as normal, but later confirmed to show retrospectively visible malignancy at subsequent screen two years later, confirmed through biopsy;
3. Contra: Current mammogram of the normal breast from women (n = 40) with a cancer reported in the opposite breast;
4. Prior-Invis: Prior mammograms of women (n = 40) who later developed visible cancer but who had no visible signs of cancer in the prior images that were reported as normal;
5. Normal: Prior mammograms of women (n = 40) reported as normal and confirmed to be cancer-free using subsequent mammograms obtained two years later. These mammograms served as a baseline for comparison to that of women diagnosed with cancer.

### Participants

Twenty-three Royal Australian & New Zealand College of Radiology (RANZCR) certified breast radiologists and breast physicians (2 breast physicians, both certified BreastScreen Australia readers) participated in the study. At the time they attended the 2017 RANZCR Breast Imaging Group (BIG) meeting. Participants self-reported their demographic information which included age, gender, years since qualification as radiologists, years since registration as breast screening radiologists, number of hours per week currently spent reading mammograms and number of cases per week. They were also asked whether they had completed any breast reading fellowship, which lasted for 3–6 months and whether they are working for Australia or New Zealand screening program. The characteristics of participants are shown in Table 5.

**Table 5:** Demographic information of the participants.

Characteristics	Participants^*^
Gender	
Female	14 (60.87%)
Male	9 (39.13%)
Age	
30–39 y	4 (17.39%)
40–49 y	6 (26.09%)
50–59 y	9 (39.13%)
60–69 y	4 (17.39%)
Whether completed a breast fellowship	
Yes	7 (30.43%)
No	16 (69.57%)
Screen reader	
Yes	20 (86.96%)
No	3 (13.04%)
Years reading mammograms	
5 or less than 5 y	4 (17.39%)
6–10 y	5 (21.74%)
11–15 y	4 (17.39%)
16–20 y	4 (17.39%)
More than 20 y	6 (26.09%)
Years since registration as breast screening radiologists	
5 or less than 5 y	7 (30.43%)
6–10 y	4 (17.39%)
11–15 y	5 (21.74%)
16–20 y	4 (17.39%)
More than 20 y	3 (13.04%)
Hours per week reading mammograms	
4 or less than 4 h	5 (21.74%)
5–10 h	13 (56.52%)
11–15 h	2 (8.70%)
16–20 h	2 (8.70%)
More than 20 h	1 (4.35%)
Number of mammograms per week	
Less than 20	4 (17.39%)
20–59	1 (4.35%)
60–100	5 (21.74%)
101–150	2 (8.70%)
151–200	4 (17.39%)
More than 200	7 (30.43%)

^*^Number of readers in each category (percentage); total number of participant was 23.

### Experimental procedure

Images were presented using a MATLAB-based (Mathworks, Natick, MA, USA) computer application, which was run on a Microsoft Surface Pro 4. For the presentation of images, Microsoft Surface Pro 4 was connected to a Philips 28-inch LED (Model 288P6LJEB) display with a resolution of 3840 × 2160 pixels and typical brightness of 300 cd/m². The original resolution of mammograms were 4915 × 5355. First, they were cropped to include only the breast area. All images were then resized so that their heights were 1944 pixels. The ambient illumination was less than 10 lux. The steps of presenting each case is shown in Fig. 1. First, a cross appeared in the centre of the screen for 500 milliseconds. This was followed by a 500 milliseconds presentation of a mammogram that readers evaluated for any appearance of abnormality. After this, a white region, the shape of the breast was presented for 500 milliseconds for masking to end visual processing of the image. This was followed by a rating interface and observers were asked to rate the image they had just seen. Radiologists were able to use the touch screen to set the slider-bar. Prior to the experiment, radiologists were shown how to use the image rating software and a pilot trial was performed to ensure that participants were familiar with the software package. Six mammograms were used for training radiologists on the use of the software.

All mammograms were presented to participants in a randomized order and were independently rated by each participant. The sequence for presenting the images was randomly generated for each reader. Each participant viewed each image and rated whether or not they would recall the patient on a scale of 0–100, with 0 indicating complete confidence that cases were normal and 100 indicating confident abnormality. No feedback was provided to the participants.

### Statistical analysis

A Kruskal–Wallis H test was used to assess whether the scores given by readers for various categories of mammograms included in the study differed significantly. The score given by the radiologists to each case was on the scale of 0–100. Before data collection, the radiologists were instructed that the score of 0 and 100 indicates complete confidence that cases are normal and abnormal respectively while the score of 50, which is the default value of the slider in the software, indicates that the mammogram has equal probability of being a normal or abnormal case.

Also, four pairwise comparisons of mean AUC values were performed to investigate whether the radiologists distinguish Normal categories from other categories at above-chance level. To do so, mammograms belonging to Normal condition served as a baseline (Negative instances), and were compared to each of the other four mammogram categories. Therefore, for each reader, comparisons were made between Cancer vs Normal, Contra vs Normal, Prior-Vis vs Normal, and Prior-Invis vs Normal. Each time 80 images were involved, 40 images of which were negative (Normal), and 40 images from one of the four other categories described above served as positive instances. ROC curves, plotting the true positive rate as a function of false positive rate, were created for each participant and for each pair. For each curve, an AUC value was calculated as a measure of the reader’s ability to distinguish positive/abnormal images from the normal images. Then, the Wilcoxon Signed Rank test was used to examine whether the AUC estimate for the readers was different from chance level (i.e. 0.5). The null hypothesis was that the median of the AUC values for all readers was 0.5. A significant difference at less than or equal to a p value of 0.05 indicates that the average reader could distinguish abnormal cases from normal ones at an above-chance level.

A Pearson correlation analysis was used to determine if radiologists who performed better in distinguishing Prior-Invis from Normal, also performed better in each of the other three comparisons. Again, a p-value less than or equal to 0.05 was used to indicate a statistically significant relationship. Given the fact that Pearson correlation analysis showed the AUC scores for different categories were correlated, we hypothesized that the variations in AUC scores corresponding to these four pairs can be reasonably described by only one latent variable, describing the overall ability of a radiologist to perceive the global gist signal. A confirmatory factor analysis with one common factor was conducted to bolster this hypothesis. We also performed the Kaiser-Meyer-Olkin (KMO) measure of sampling adequacy to investigate whether the assumption of factorability is reasonable. The KMO measure above 0.6 is recommended for factor analysis. The KMO measure for the data presented in Table 1 was 0.82. This value suggests the data will support factor analysis. To examine the goodness of fit for this unidimensional model, chi-square (χ^2^) was used. Its corresponding p-value shows the magnitude of mis-fit between the model and the data. Since the p-value were markedly greater than 0.05, we conclude that there is no support for a significant difference between the data and the specified unidimensional model.

We also investigated if the gist response was related to the lesion type, breast density, and lesion position. To do so, by employing the Kruskal Wallis H-test, we investigated if the average gist response varied across different BIRADS density categories, lesion types, and lesion position for each category of abnormal cases, i.e. Cancer, Prior_Vis, Contra, and Prior_Invis as well as all abnormal cases when combined together. P-value of 0.05 or less was considered significant. For two continues variables, i.e. lesion size and lesion distance to the center point, two sets of statistical analyses performed. First, the images were categorized by using the median value of each variable as the threshold. Similar to other categorical variables, Kruskal Wallis H-test was utilized. One might argue that the categorization of variables could eliminate the dependency between the continuous variables and the gist response. Therefore, we also used the HSIC for testing the independence of the mean gist responses and the lesion size as well as lesion distance to the center point. The HSIC indicated whether the mean gist response was independent from the lesion size and lesion distance.

### Data availability

All relevant data are available from the authors.
